# Extracts and compounds with anti-diabetic complications and anti-cancer activity from *Castanea mollissina* Blume (Chinese chestnut)

**DOI:** 10.1186/1472-6882-14-422

**Published:** 2014-10-28

**Authors:** Lin Zhang, Hui-yuan Gao, Masaki Baba, Yoshihito Okada, Toru Okuyama, Li-jun Wu, Li-bin Zhan

**Affiliations:** Academy of Integrative Medicine, Dalian Medical University, No. 9, Western Section of South Road in Lvshun, Dalian, 116044 China; Key Laboratory of Structure-Based Drug Design & Discovery of Ministry of Education, Shenyang Pharmaceutical University, No.103, Wenhua Road, Shenhe District, Shenyang, 110016 China; Department of Natural Medicine and Phytochemistry, Meiji Pharmaceutical University, No. 2-522-1, Noshio, Kiyose, Tokyo, 204-8588 Japan; Department of Traditional Chinese Medicine, The Second Affiliated Hospital, Dalian Medical University, Dalian, 116023 China

**Keywords:** Anti-diabetic complications, Anti-cancer, *Castanea mollissina* Blume, Phenolic acids, Flavonoids

## Abstract

**Background:**

*Castanea mollissima* Blume (Chinese chestnut), as a food product is known for its various nutrients and functional values to the human health. The present study was carried out to analyze the anti-diabetic complications and anti-cancer activities of the bioactive compounds present in *C. mollissima*.

**Methods:**

The kernels (CK), shells (CS) and involucres (CI) parts of *C.* Blume were extracted with 90% alcohol. The water suspension of these dried alcohol extracts were extracted using EtOAc and *n*-BuOH successively. The *n*-BuOH fraction of CI (CI-B) was isolated by silica gel column, Sephadex LH 20 column and preparative HPLC. The isolated compounds were identified by ^1^H-NMR, ^13^C-NMR, HMBC, HMQC and ESI-Q-TOF MS, All the fractions and compounds isolated were evaluated on human recombinant aldose reductase (HR-AR) assay, advanced glycation end products (AGEs) formation assay and human COLO 320 DM colon cancer cells inhibitory assay.

**Results:**

CI-B was found to show a significant inhibitory effect in above biological screenings. Six flavonoids and three polyphenolic acids were obtained from CI-B. They were identified as kaempferol (1), kaempferol-3-*O*-[6''-*O*-(*E*)-*p*-coumaroyl]-*β*-D-glucopyranoside (2), kaempferol-3-*O*-[6''-*O*-(*E*)-*p*-coumaroyl]-*β*-D-galactopyranoside (3), kaempferol-3-*O*-[2''-*O*-(*E*)-*p*-coumaroyl]-*β*-D-glucopyranoside (4), kaempferol-3-*O*-[2", 6"-di-*O*-(*E*)-*p*-coumaroyl]-*β*-D-glucopyranoside (5) and kaempferol-3-*O*-[2", 6"-di-*O*-(*E*)-*p*-coumaroyl]-*β*-D-galactopyranoside (6), casuariin (7), casuarinin (8) and castalagin (9). Compounds 2–9 were found to show higher activity than quercetin (positive control) in the AR assay. Compounds 3–6, 8, and 9 showed stronger inhibitory effects than amino guanidine (positive control) on AGEs production. Compounds 4–6, 7, and 8 showed much higher cytotoxic activity than 5-fluorouracil (positive control) against the human COLO 320 DM colon cancer cells.

**Conclusions:**

Our results suggest that flavonoids and polyphenolic acids possesses anti-diabetes complications and anti-cancer properties, and they were presumed to be the bioactive components of *Castanea mollissima* Blume.

**Electronic supplementary material:**

The online version of this article (doi:10.1186/1472-6882-14-422) contains supplementary material, which is available to authorized users.

## Background

*Castanea mollissima* Blume (family Fagaceae), with the Chinese name “Ban li” (chestnut) is widely distributed in Asian areas, such as China, Korea and Viet Nam. Chestnut fruits are highly regarded and widely consumed throughout Asia, Europe and America. Various commercial forms are available. Ban li is not only used as a food product for its various nutrients, but also used as a traditional Chinese medicine, including the flowers, leaves, and twigs to have been used to treat gastroenteritis, bronchitis and regurgitation for hundreds years [1]. It is no doubt that chestnuts have considerable potential value as functional foods [2].

To increase and find much more functional values for the Chinese chestnut, our previous work on the chemical constituents and bioactivity of it resulted in the isolation of many flavonoids and phenolic acids [3–6]. In the continuous assessing its bioactivity and finding more active agents, the anti-diabetic complications and anti-cancer activity of all fractions of alcohol extracts of kernels (CK), shells (CS) and involucres (CI) were evaluated on human recombinant aldose reductase (HR-AR) assay, advanced glycation end products (AGEs) formation assay and human COLO 320 DM colon cancer cells inhibitory assay in the present work, respectively.

Diabetes, as a complex metabolic disorder caused by insulin insufficiency and/or insulin dysfunction, is characterized by aberrant blood glucose and insulin levels [7]. Diabetic complications, including retinopathy, neuropathy, nephropathy, and arteriosclerosis are considered as risk factors for morbidity and death. Moreover, the diabetic patients are also susceptible to many diseases, including the cancer. For example, colorectal cancer which is the third leading cause of cancer-related death [8], is much easier happened in diabetic patient [9]. Many studies on chestnut seeds and other parts of this plant emphasised on the anti-oxidant property [2, 10], while, the present work were to value their anti-diabetes complications and against the connecting cancer activity, also to find the part with much functional values.

## Methods

### Plant material

The kernel, shells and involucres parts of *C. mollissima* were collected respectively in September, 2005 in Qianxi County of Hebei province, and identified by Professor Sun Qishi (College of Traditional Chinese Medicine, Shenyang Pharmaceutical University). The voucher specimens were deposited at the Key Laboratory of Structure-Based Drug Design & Discovery of Ministry of Education (No.ZB2005-026-028).

### Chemical and reagents

Dibasic sodium phosphate, sodium dihydrogen phosphate, D,L-glyceraldehyde, human recombinant aldose reductase (HR-AR), AG, quercetin, critric acid monohydrate, natrium carbonicum, sodium azide, gelatin and sulphuric acid were purchased from Wako Pure Chemical Industries, Ltd. (Osaka, Japan). Sodium bicarbonate, sodium chloride, potassium dihydrogen phosphate and ethanol were supplied by Nacalai Inc. (Kyoto, Japan). Tween 20, bovine serum albumin, glucose, *O*-phenylenediamine dihydrochloride, phosphate buffered saline, fetal bovine serum (FBS), steroyl myristoyl phosphatidylcholine glycine (SMPC Gly), 3-(4, 5-dimethylthazol-2-yl)-2, 5-diphenyl tetrazolium bromidetetrazolium salt (MTT) were purchased from Sigma-Aldrich company, Ltd. (St. Louis, MO, U.S.A.). NADPH was provided by Oriental Yeast Co., Ltd. (Tokyo, Japan). Anti-AGE antibody and goat anti-mouse IgG, HRP conjugate-secondary antibody Millipore (Merck U.S.A) were purchased from Transgenic Inc. (Hyogo, Japan). Silica gel 60 F_254_ TLC plates (Merck, U.S.A), silica gel 60 N (100–200 μm) and ODS were purchased from Kanto Chemical (Tokyo, Japan). DMSO was purchased from Wako Pure Chemical Industries, Ltd (Japan). Kaempferol-3-*O*-*β*-D-glucopyranoside was isolated from liquorice, and was identified by NMR and MS data [11].

### Instrument

Analysis HPLC was carried out on Waters 510 with Waters 484 detector (waters, USA) using a kromasil C18 column (4.6 × 200 mm, Rainbow). Preparative HPLC was carried out on JASCO PU-2087 with JASCO PU-2075 detector (JASCO, Japan) using a HiQ sil C18 column (10 × 200 mm, YATECH). ESI-MS spectra were recorded on a Bruker esquire 2000 mass spectrometer. NMR spectra (^1^H-, ^13^C-NMR, HMBC and NOESY) were measured and recorded on Bruker AVANCE 600 (Bruker, Newark, DE) in DMSO using TMS as internal standard. Infrared (IR) spectra were measured on a Fourier transform infrared spectrometer (IFS-55; Bruker).

### HR-AR assay

Aldose reductase activities of samples were assayed spectrophotometrically by determining the decrease in NADPH concentration at 340 nm in a UV-2201 Pharma Spec UV–vis spectrophotometer (Shimadzu, Japan). The reaction mixture contained 0.14 M phosphate buffer pH 6.2, 700 μL; 0.15 mM NADPH, 100 μL; 3 × 10^-2^ units/mL AR, 100 μL; 10 mM D,L-glyceraldehyde, 3.3 mg/mL extracts or 1 mg/mL compounds in DMSO, 3 μL, in a total volume of 1 mL with a final concentration of 10 μg/mL for extracts and 3.3 μg/mL for compounds. The reference blank contained all of the above reagents, and buffer instead of AR, to correct for nonspecific reduction of NADPH. The control had only the sample solvent instead of the sample to correct for reduction of NADPH without inhibitor of AR (ARI). The reaction was initiated by the addition of substrate, and it was monitored spectrophotometrically for 3 min. All values were averages of three independent experiments [12]. The inhibition ratio of AR was calculated by following equation:


### Inhibit formation of AGEs assay

The AGEs reaction solution (200 μL) was composed of 16 mg/mL bovine serum albumin, 50 μL; 0.1 M sodium phosphate buffer (pH 7.4), 96 μL; 144 mg/mL glucose, 50 μL; 0.5 mg/mL sample in DMSO, 4 μL with a final concentration of 10 μg/mL. The sample blank, control solvent and blank solvent contained all of the above reagents, except sample solvent with buffer instead of glucose in the sample blank, sample solvent instead of sample in the control solvent, and sample solvent instead of sample, buffer instead of glucose in the blank solvent. After incubating at 37°C for 7 days, the amounts of AGE products were determined by enzyme-linked immunosorbent assay (ELISA) [13]. Noncompetitive ELISA assays were performed at room temperature. Each well was incubated for 1 h with 0.1 mL of an AGE sample to be tested or its corresponding control sample in 50 mM carbonate buffer (pH 9.7) and washed three times with washing buffer (phosphate-buffered saline containing 0.05% Tween 20). Each well was then blocked for 1 h with 0.2 mL of 2.5% gelatin in 5 mM carbonate buffer (pH 9.7). Each well was washed three times with washing buffer and incubated for 1 h with 0.1 mL of anti-AGE antibody (50 ng/mL). Wells were then washed three times with washing buffer and incubated for 1 h with 0.1 mL of goat anti-mouse IgG, HRP conjugate-secondary antibody (Millipore, Merck) and then washed three times, followed by reaction with *o*-phenylenediamine dihydrochloride and hydrogen peroxide mixture. The reaction was terminated by adding 1 M sulfuric acid, and the absorbance at 492 nm was read on a micro-plate reader (MPR A4i II TOSOH). All values are averages of three independent experiments, each done in triplicate [14]. The percentage inhibition of AGE production was calculated by the following equation:


### Cytotoxic assay

Human COLO 320 DM colon cancer cells (passage-10) were maintained in RPMI-1640 medium supplemented with 10% FBS and SMPC Gly at 37°C in an incubator. The cytotoxicity was determined by the MTT method. Briefly, exponentially growing cells, 100 μl, were attached at 5 × 10^4^ cells/well, in 96-well plates, and the cellular viability was determined after 24 h, 48 h, and 72 h administration of the extractions (100 μg/mL) 100 μL. Cells were incubated with MTT tetrazolium salt for 1 h at 37°C, and the formation of formazan was measured by a microplate reader (MPR A4iIITOSOH). All values are averages of two independent experiments, each done in triplicate. The percentage inhibition of cell growth was calculated by the following equation:


Where A0 is the absorbance of the control at 500 nm after incubations, and A1 is the absorbance in the presence of the samples. The study was performed in accordance with the Declaration of Helsinki.

### Extraction and isolation

Air-dried three parts of kernels 6 kg (CK), shells 5 kg (CS) and involucres 10 kg (CI) of were *C. mollissima* crushed and extracted with ethanol-water (90:10, v/v) (CK: 36 L; CS: 30 L and CI: 60 L) for three times (for 2 h × 3) respectively, then, the solutions were concentrated under reduced pressure using a rotary evaporator less than 40°C, to give extracts (CK 953.2 g, 15.89%; CS 257.4 g, 5.15%; CI 305.6 g, 3.06%), respectively. The extracts were suspended in distilled water (CK: 6 L, CS: 5 L, and CI: 10 L) and partitioned successively with the same volume EtOAc and *n*-BuOH successively. After removing organic solvents, three EtOAC soluble fractions CK-A (30.2 g, 0.50%), CS-A (35.9 g, 0.72%), CI-A (95.7 g, 0.96%) and three *n*-BuOH soluble fractions CK-B (61.5 g, 1.03%), CS-B (21.8 g, 0.44%), CI-B (62.1 g, 0.62%), along with three aqueous parts CK-W (811.9 g, 13.53%), CS-W (140.6 g, 2.81%), CI-W (125.9 g, 1.26%) were obtained. Each part was vacuum-packed and stored at -20°C until use.

**Figure 1:**
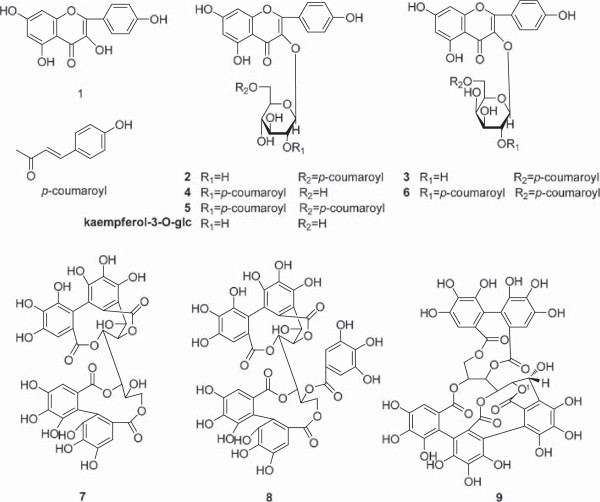
**The structures of compounds 1–9.**

Part of CI-B (60 g) was taken and mixed with 180 g silica then applied to a 85 cm × 10 cm (inside diameter) silica gel column and eluted with different ratios of EtOAc and MeOH (10:1, 10:2, 10:4, 10:6, 10:8, 1:1, 1:5, 0:1) to give 10 fractions based on the results of TLC experiment. Fraction 2 (9.75 g) eluted with EtOAc-MeOH (10:1) was further separated into 12 sub-fractions by a 60 cm × 7 cm (inside diameter) silica gel column based on the results of TLC experiment. Sub-fraction 7 (1.25 g) eluted with Hexane-EtOAc (1:3) was purified by preparative HPLC with MeOH-H_2_O (60:40 v/v, 1.5 mL/min) on a C_18_-MS-II-waters column (10 × 250 mm) to yield compound **1** (30 mg). Subfraction 9 (0.87 g) eluted with Hexane-EtOAc (3:1) was purified by a 40 cm × 6 cm (inside diameter) ODS gel column with MeOH-H_2_O (30:70, 40:60, 50:50, 60:40, 100:0 v/v) and preparative HPLC with MeOH-H_2_O (55:45 v/v, 1.5 mL/min) on a C_18_-MS-waters column (10 × 250 mm) to yield compound **5** (30 mg) and compound **6** (15 mg). Sub-fraction 10 (1.50 g) eluted with Hexane-EtOAc (6:1) was purified by preparative HPLC with MeOH-H_2_O (55:45 v/v) on a C_18_-MS-waters column (10 × 250 mm) to yield compounds **2** (7 mg), **3** (30 mg) and **4** (25 mg). Fraction 3 (26.0 g) eluted with EtOAc-MeOH (10:1) was further separated into 8 sub-fractions by a 80 cm × 9 cm (inside diameter) silica gel column. Compounds **7**–**9** was obtained from sub-fraction 5 (5.36 g) which was eluted with Hexane-EtOAc (6:1) by a 50 cm × 7 cm (inside diameter) ODS gel column with MeOH-H_2_O (30:70, 40:60, 50:50, 60:40, 100:0 v/v) and an 80 cm × 4 cm (inside diameter) Sephadex LH-20 column with MeOH-H_2_O (50:50 v/v). Structures of compounds **1**–**9** were shown in Figure 1.

### Hydrolysis of compound 6

Compound **6** (1.0 mg) was dissolved in MeOH (9 mL) containing HCl (1 mL) and refluxed on a heated (80°C) water bath for 3 h. After cooling, the reaction mixture was concentrated and dried under reduced pressure, then analyzed by TLC using the solvent system CHCl_3_–MeOH–H_2_O (20:10:1) for identification of the complete reaction. The dried residues dissolved in EtOAc, and partitioned with water. The water solution was analyzed and subjected to HPLC with optical detector using a Kaseisorb LC NH_2_-60-5 column (4.6 mm i.d. × 250 nm) with CH_3_CN–H_2_O (v:v = 85:15, Flow: 0.8 mL/min) as the mobile phase. Identification of D-galactose was carried out by comparison of the retention time (13.2 min) and OD (+) with authentic samples.

## Results

### Structural determination of compound 6

**Figure 2:**
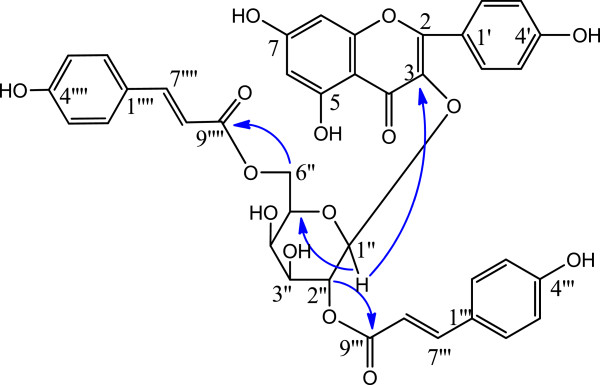
**Key HMBC correlations of compound 6.**

Kaempferol-3-*O*-[2", 6"-di-*O*-(*E*)-*p*-coumaroyl]-*β*-D-galactopyranoside (**6**) was obtained as a yellow powder with a negative optical rotation. Its structure identification was achieved by mass and NMR spectroscopy (Additional files 1, 2, 3, 4, 5, 6 and 7), including two dimensional correlated NMR (NOESY, HMQC, HMBC). Its molecular formula was established as C_39_H_32_O_15_ by means of ESI-Q-TOF MS which showed an [M + H]^+^ ion peak at *m/z* 741.1824 (calcd 741.1819 for C_39_H_33_O_15_). ^1^H- and ^13^C NMR spectral data of **6** (Table 1) showed the presence of a kaempferol residue, two *p*-coumaroyl groups and one sugar unit. The HMBC spectrum (Figure 2) clarified the attachment of the sugar group at C-3 position based on a correlation between the anomeric proton signal at *δ*_H_ 5.54 (d, *J* = 7.6) with C-3 (104.65). The attachments for two *p*-coumaroyl groups at C-2″ and C-6″ were based on correlations between *δ*_H_ 5.38 (dd, *J* = 9.6, 8.0, H-2″) with *δ*_C_ 168.23 (C-9″′), and, *δ*_H_ 4.19, 4.36 (dd, 11.6, H-6") with *δ*_C_ 168.21 (C-9""), respectively. The *trans* form configurations for their double bonds at C-7''', 8''' and C-7'''', 8'''' positions were confirmed by the coupling constant values 15.6 Hz for H-7''' and H-7'''' [15]. The sugar unit was identified as galactose after the sample was dealt with the acid hydrolysis and compared the t_R_ with the authentic sample by HPLC analysis using a Kaseisorb LC NH_2_-60-5 column (4.6 mm i.d. × 250 nm), CH_3_CN–H_2_O as the mobile phase. Compound **6** was identified as kaempferol-3-*O*-[2", 6"-di-*O*-(*E*)-*p*-coumaroyl]-*β*-D-galactopyranoside at finally.

**Table 1:** **NMR spectra data of compound 6 in CD**_**3**_ **OD**

No.	^1^H-NMR	^13^C-NMR	HMBC
2		158.17 s	
3		134.58 s	
4		179.00 s	
5		162.69 s	
6	6.06 (1H, br.s)	99.79 d	5, 7, 8, 10
7		165.32 s	
8	6.25 (1H, d, 1.6)	94.54 d	6, 7, 9, 10
9		157.96 s	
10		105.55 s	
1′		122.52 s	
2′, 6′	7.96 (2H, d, 8.8)	131.95 d	2, 4′
3′, 5′	6.87 (2H, d, 8.8)	116.10 d	4′, 1′
4′		161.13 s	
1′′	5.54 (1H, d, 8.0 )	100.85 d	3, 2′′
2′′	5.38 (1H, dd, 9.6,8.0)	73.98 d	1′′, 3′′, 9′′′
3′′	3.82 (1H, m)	73.13 d	2′′
4′′	3.88 (1H, m)	70.53 d	2′′
5′′	3.85 (1H, m)	74.90 d	
6′′	4.19 (1H, dd, 11.6)	64.15 t	9′′′′
	4.36 (1H, dd, 11.6)		
1′′′		127.09 s	
2′′′, 6′′′	7.46 (2H, d, 8.4)	131.00 d	4′′′, 7′′′
3′′′, 5′′′	6.79 (2H, d, 8.4)	116.59 d	1′′′, 4′′′
4′′′		160.94 s	
7′′′	7.69 (1H, d, 15.6)	146.68 d	6′′′, 8′′′, 9′′′
8′′′	6.40 (1H, d, 15.6)	115.12 d	1′′′, 9′′′
9′′′		168.23 s	
1′′′′		126.87 s	
2′′′′, 6′′′′	7.27 (2H, d, 8.4)	130.92 d	4′′′′, 7′′′′
3′′′′, 5′′′′	6.79 (2H, d, 8.4)	116.59 d	1′′′′, 4′′′′
4′′′′		160.87 s	
7′′′′	7.40 (1H, d, 15.6)	146.35 d	6′′′′, 8′′′′, 9′′′′
8′′′′	6.05 (1H, d, 15.6)	114.43 d	9′′′′
9′′′′		168.21 s

### Effects of fractions and compounds in HR-AR, AGEs and Cytotoxic assay

**Figure 3:**
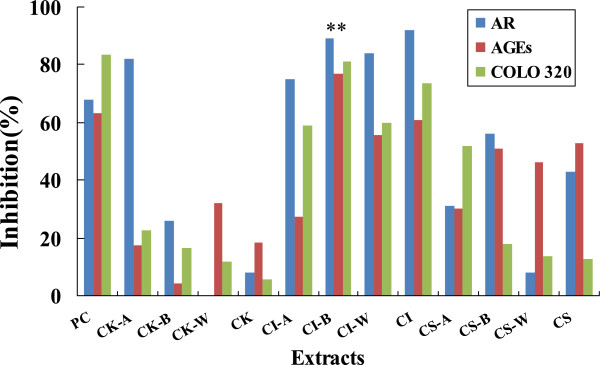
**Inhibition ratio of all parts of the kernel (CK-A, CK-B, CK-W, CK), shell (CS-A, CS-B, CS-W, CS) and involucre (CI-A, CI-B, CI-W, CI) (10 μg/mL) in the AR assay, AGEs production and COLO 320DM proliferation.** Positive Control (PC): Quercetin (3.3 μg/mL) for AR assay, AG (amino guanidine, 0.2 mM) for AGEs production and 5-Fu (5-fluorouracil, 50 μg/mL) for COLO 320DM proliferation, respectively.

All crude materials and soluble fractions (EtOAc, *n*-BuOH and water part) of kernels (CK, CK-A, CK-B, CK-W), shells (CS, CS-A, CS-B, CS-W) and involucres (CI, CI-A, CI-B, CI-W) were evaluated on AR, AGEs and the growth inhibitory activity aginst human COLO 320 DM colon cancer cells. Among these fractions, the *n*-BuOH soluble fraction of involucres (CI-B) was found to be significantly more active than others (Figure 3), and its inhibitory rates were 88.6 ± 2.3% in AR, 77.0 ± 5.8% in AGEs, and 81.3 ± 3.7% in cytotoxic assay respectively. Effective compounds from CI-B were further studied. Six flavonoids, kaempferol (**1**), kaempferol-3-*O*-[6"-*O*-(*E*)-*p*-coumaroyl]-β-D-glucopyranoside (**2**), kaempferol-3-*O*-[6"-*O*-(*E*)-*p*-coumaroyl]-*β*-D-galatopyranoside (**3**), kaempferol-3-*O*-[2"-*O*-(*E*)-*p*-coumaroyl]-*β*-D-glucopyranoside (**4**), kaempferol-3-*O*-[2", 6"-di-*O*-(*E*)-*p*-coumaroyl]-*β*-D-glucopyranoside (**5**), kaempferol-3-*O*-[2", 6"-di-*O*-(*E*)-*p*-coumaroyl]-*β*-D-galactopyranoside (**6**), and three polyphenolic acids, casuariin (**7**), casuarinin (**8**), and castalagin (**9**) were obtained. Structures of compounds (see Figure 1) were elucidated by the spectroscopic methods, including UV, ESI-MS, NMR experiments, and comparing their spectral data with reported in the references [3, 16, 17]. The inhibitory activities of compounds **1**–**9** were shown in Table 2.

**Table 2:** **AR, AGEs and COLO 320 inhibitory effects of Compounds 1–9 and kaempferol-3-O-β-D-glucopyranoside compared with Qu, AG and 5-Fu**

Compounds	IC _50_ (μM)		
	AR	AGEs	COLO 320
1	14.83 ± 0.28^△^	57.06 ± 7.54	>100
2	2.39 ± 0.20***	28.73 ± 2.96	24.10 ± 3.26
3	1.83 ± 0.02***	8.48 ± 1.43*	22.56 ± 4.51^△^
4	1.22 ± 0.06***	5.58 ± 0.48^***^	3.53 ± 0.42^**^
5	0.43 ± 0.06***	3.27 ± 0.09^***^	2.86 ± 0.39^**^
6	0.63 ± 0.12***	0.75 ± 0.21^***^	2.43 ± 1.01^**^
kaempferol-3-*O*-glc	10.40 ± 0.18**	42.64 ± 3.73	58.84 ± 3.26
7	6.33 ± 1.10**	12.69 ± 5.17^△△^	10.07 ± 1.84^*^
8	0.46 ± 0.01***	4.43 ± 0.36^***^	5.49 ± 0.23^*^
9	1.73 ± 0.14***	5.21 ± 0.75^***^	3.67 ± 0.09^**^
Qu	12.35 ± 1.06		
AG		10.68 ± 1.06	
5-Fu			8.79 ± 2.57

Data represent mean ± S.E.M. (n=3). IC_50_ of Compounds< IC_50_ of Positive Control (Qu, AG, 5-Fu): ^***^ *p* < 0.01 *vs* Positive Control, ^**^ *p* < 0.05 *vs* Positive Control, ^*^ *p* >0.05 *vs* Positive Control; IC_50_ of Compounds> IC_50_ of Positive Control (Qu, AG, 5-Fu): ^△△^ *p* > 0.05 *vs* Positive Control. ^△^ *p* > 0.01 *vs* Positive Control.

### Physicochemical data of compounds 2–9

kaempferol-3-*O*-[6"-*O*-(*E*)-*p*-coumaroyl]-*β*-D-glucopyranoside

**(2).** Yellow powder; m.p.223-225°C; [α]_D_^23^ -39.0° (c 0.05, MeOH). UV λ_max_ (MeOH) nm (logϵ): 316 (2.25). FAB-MS m/z: 595 [M + H]^+^ and 593 [M-H]^-^. ^1^H-NMR and ^13^C-NMR spectra data see Table 3.

**Table 3:** ^**1**^ **H-NMR and** ^**13**^ **C-NMR data of compounds 2-5**

NO.	2		3		4		5	
	^1^H-NMR ^1^	^13^C-NMR ^1^	^1^H-NMR ^1^	^13^C-NMR ^1^	^1^H-NMR ^2^	^13^C-NMR ^2^	^1^H-NMR ^2^	^13^C-NMR ^2^
2		156.4		156.4		158.1		158.7
3		133.0		133.0		134.6		134.2
4		177.4		177.4		178.8		178.8
5		156.3		156.3		162.8		162.7
6	6.15 (1H, d, 1.9)	98.7	6.15 (1H, d, 1.9)	98.7	6.05 (1H, br.s)	100.4	6.05 (1H, br.s)	99.7
7		164.1		164.1		165.4		165.3
8	6.39 (1H, d, 1.9)	93.5	6.39 (1H, d, 1.9)	93.5	6.24 (1H, br.s)	94.5	6.25 (1H, br.s)	94.6
9		161.1		161.1		158.0		158.0
10		103.9		103.9		105.7		105.6
1′		120.7		120.7		122.6		122.7
2′, 6′	7.99 (2H, d, 9.2)	130.8	7.99 (2H, d, 9.2)	130.8	7.89 (2H, d, 8.8)	132.0	7.95 (2H, d, 8.8)	131.9
3′, 5′	6.86 (2H, d, 9.2)	115.1	6.86 (2H, d, 9.2)	115.1	6.78 (2H, d, 8.8)	116.0	6.86 (2H, d, 8.8)	115.9
4′		160.0		160.0		161.2		160.9
1′′	5.45 (1H, d, 7.7)	100.9	5.45 (1H, d, 7.7)	100.9	5.57 (1H, d, 8.0)	99.6	5.65 (1H, d, 8.4 )	100.3
2′′	3.28 (1H, d, 7.7)	74.2	3.28 (1H, d, 7.7)	74.2	4.92 (1H, d, 8.0)	75.7	5.09 (1H, dd, 8.8,8.4)	75.6
3′′	3.25 (1H, d, 7.7)	76.2	3.25 (1H, d, 7.7)	76.2	3.48 m	76.2	3.71 (1H, dd, 8.8,8.8)	76.1
4′′	3.18 (1H, m)	69.9	3.18 (1H, m)	69.9	3.30 m (1H, d, 8.8)	71.5	3.44 (1H, dd, 8.8,8.8)	71.9
5′′	3.38 (1H, m)	74.1	3.38 (1H, m)	74.1	3.21 m	78.6	3.59 (1H, m)	75.8
6′′	4.28 (1H, dd, 12.0, 2.0)	63.0	4.28 (1H, dd, 12.0, 2.0)	63.0	3.67 m	62.5	4.23 (1H, br.d, 10.8)	64.1
	4.03 (1H, dd, 12.0, 2.0)		4.03 (1H, dd, 12.0, 2.0)		3.46 m		4.37 (1H, br.d, 10.8)	
1′′′		124.9		124.9		127.1		126.9
2′′′, 6′′′	7.37 (2H, d, 8.5)	130.1	7.37 (2H, d, 8.5)	130.1	7.34 (2H, d, 8.4)	131.0	7.30 (2H, d, 8.8)	131.0
3′′′, 5′′′	6.79 (2H, d, 8.5)	115.6	6.79 (2H, d, 8.5)	115.6	6.70 (2H, d, 8.4)	116.6	6.80 (2H, d, 8.8)	116.6
4′′′		159.8		159.8		160.9		160.8
7′′′	7.34 (1H, d, 15.8)	144.6	7.34 (1H, d, 15.8)	144.6	7.55 (1H, d, 16.0)	146.6	7.40 (1H, d, 15.6)	146.3
8′′′	6.11 (1H, d, 15.8)	113.6	6.11 (1H, d, 15.8)	113.6	6.25 (1H, d, 16.0)	115.0	6.07 (1H, d, 15.6)	114.5
9′′′		166.1		166.1		168.1		168.5
1′′′′								126.9
2′′′′, 6′′′′							7.48 (2H, d, 8.8)	131.0
3′′′′, 5′′′′							6.80 (2H, d, 8.8)	116.6
4′′′′								160.9
7′′′′							7.72 (1H, d, 16.0)	146.8
8′′′′							6.44 (1H, d, 16.0)	115.1
9′′′′								168.2

kaempferol-3-*O*-[6''-*O*-(*E*)-*p*-coumaroyl]-*β*-D-galatopyranoside

(**3**). Yellow powder (40.0 mg). m.p.245-247°C. [α]_D_^22^ -43.0° (c 0.05, MeOH). UV λ_max_ (MeOH) nm (logϵ): 318 (2.09). FAB-MS m/z: 595 [M + H]^+^ and 593 [M-H]^-^. ^1^H-NMR and ^13^C-NMR spectra data see Table 3.

kaempferol-3-*O*-[2''-*O*-(*E*)-*p*-coumaroyl]-*β*-D-glucopyranoside

(**4**). Yellow powder. m.p.245-247°C. [α]_D_^20^ -74.1° (c 0.13, MeOH). UV λ_max_ (MeOH) nm (logϵ): 316 (2.74). FAB-MS m/z: 595 [M + H]^+^ and 593 [M-H]^-^. ^1^H-NMR and ^13^C-NMR spectra data see Table 3.

kaempferol-3-*O*-[2", 6"-di-*O*-(*E*)-*p*-coumaroyl]-*β*-D-glucopyranoside

(**5**). Yellow powder. m.p.196-199°C. [α]_D_^22^ -119.6° (c 0.8, MeOH). UV λ_max_ (MeOH) nm (logϵ): 330 (2.38). FAB-MS m/z: 741 [M + H]^+^. ^1^H-NMR and ^13^C-NMR spectra data see Table 3.

kaempferol-3-*O*-[2", 6"-di-*O*-(*E*)-*p*-coumaroyl]-*β*-D-galactopyranoside

(**6**). Yellow powder. m.p.200-203°C. [α]_D_^22^ -62.2° (c 0.1, MeOH). UVλ_max_ (MeOH) nm (logϵ): 323 (2.51); FAB-MS m/z: 741 [M + H]^+^, 739 [M-H]^-^ and 763 [M + Na]^+^; ESI-Q-TOF MS *m/z* 741.1824 [M + H]^+^ (calcd 741.1819 for C_39_H_33_O_15_). ^1^H-NMR, ^13^C-NMR and 2D-NMR spectra data see Table 1.

casuariin

(**7**). Off-white amorphous powder. m.p.245-248°C. FAB-MS m/z: 783 [M-H]^-^. ^1^H-NMR ((CD_3_)_2_CO + D_2_O) δ: 6.81 (1H, s), 6.62(1H, s), 6.47 (1H, s), 5.64 (1H, d, J = 4.8 Hz), 4.72 (1H, m), 5.51 (1H, m), 5.13 (1H, dd, J = 8.4, 3.2 Hz), 4.18 (1H, m), 3.93 (1H, d, J = 12.0 Hz), 4.72 (1H, d, J = 11.8 Hz). ^13^C-NMR ((CD_3_)_2_CO + D_2_O) δ: 115.9 (s), 115.6 (s), 115.6 (s), 114.5 (s), 126.8 (s), 126.5 (s), 124.6 (s), 119.4 (s), 116.6 (d), 108.2 (d), 106.6 (d), 104.8 (d), 145.5 (s), 145.2 (s), 144.7 (s), 144.6 (s), 143.9 (s), 143.8 (s), 143.2 (s), 143.0 (s), 138.3 (s), 136.2 (s), 135.2 (s), 134.4 (s), 169.8 (s), 169.3 (s), 168.3 (s), 165.3 (s), 66.6 (d), 76.6 (d), 70.3 (d), 76.5 (d), 67.8 (d), 67.9 (q).

casuarinin

(**8**). Off-white amorphous powder. m.p.244-246°C. FAB-MS m/z: 935 [M-H]^-^. ^1^H-NMR ((CD_3_)_2_C O + D_2_O) δ: 6.91 (1H, s), 6.64 (1H, s), 6.57 (1H, s), 7.16 (2H, s), 5.70 (1H, d, J = 4.8 Hz), 4.73 (1H, m), 5.47 (1H, m), 5.52 (1H, d, J = 8.8 Hz), 5.40 (1H, m), 4.20 (1H, d, J = 13.2 Hz), 4.89 (1H, m). ^13^C-NMR ((CD_3_)_2_CO + D_2_O) δ: 115.6 (s), 115.4 (s), 115.3 (s), 114.3 (s), 126.3 (s), 125.8 (s), 123.8 (s), 119.1 (s), 117.0 (s), 108.2 (d), 106.7 (d), 105.0 (d), 145.2 (s), 145.1 (s), 144.6 (s), 144.6 (s), 143.9 (s), 143.2 (s), 142.7 (s), 138.3 (s), 136.4 (s), 135.3 (s), 134.3 (s), 169.2 (s), 169.1 (s), 168.4 (s), 165.6 (s), 119.6 (s), 109.8 (d), 139.0 (s), 166.0 (s), 66.1 (d), 76.5 (d), 69.2 (d), 73.6 (d), 70.5 (d), 64.3 (q).

castalagin

(**9**). Off-white amorphous powder. m.p.243-245°C. FAB-MS m/z: 935 [M + H]^+^ and 933 [M-H]^-^. ^1^H-NMR((CD_3_)_2_CO + D_2_O) δ: 6.66 (1H, s), 6.65 (1H, s), 6.52 (1H, s), 5.57 (1H, d, J =4.4 Hz), 4.97 (1H, m), 4.92 (1H, m), 5.04 (1H, t, J = 7.2 Hz), 5.44 (1H, d, J = 7.6 Hz), 3.94 (1H, d, J = 12.8), 4.80 (1H, m). ^13^C-NMR ((CD_3_)_2_CO + D_2_O) δ: 121.3 (s), 124.4 (s), 124.4 (s), 125.8 (s), 126.9 (s), 107.2 (d), 107.9 (d), 108.4 (d), 115.7 (s), 115.5 (s), 146.1 (s), 144.9 (s), 144.7 (s), 144.6 (s), 144.2 (s), 144.0 (s), 143.9 (s), 143.8 (s), 143.6 (s), 143.1 (s), 137.5 (s), 136.2 (s), 136.0 (s), 135.4 (s), 134.5 (s), 112.4 (s), 113.9 (s), 113.9 (s), 114.5 (s), 115.4 (s), 168.9 (s), 166.9 (s), 166.2 (s), 165.1 (s), 164.7 (s), 66.0 (d), 73.8 (d), 66.6 (d), 68.9 (d), 70.9 (d), 65.2 (q).

## Discussion

Flavonoids **1**–**5** and compound kaempferol-3-*O*-*β*-D-glucopyranoside were reported to have hypolipidemic, anti-oxidant, anti-inflammatory, analgesic, and anti-aging activities [18]. The relationship between their structures and the efficiency was discussed. The presence of *ortho*-hydroxy group at B-ring, the double bond at C2–C3 for C-ring, and the presence of C_7_-OH are usually listed as important conditions for high AR inhibitory effects. Accordingly, the double bond of C2–C3, *ortho*- or *meta*-hydroxy groups in B ring, or a glucose unit are associated with the enhanced cytotoxicity [19]. According to the data for compounds **1**–**6,** the presence of *p*-coumaroyl groups would enhance the inhibitory activity in three assays, therein, flavonoids with more *p*-coumaroyl groups in structure are deemed to be the promising anti-diabetes complications and anti-cancer agents.

Compounds **7**, **8** and **9**, as polyphenolic derivatives showed remarkable inhibitory effects in three bioactive systems, and the increasing number of galloyl groups in the structure could increase their activity. Some studies found that polyphenolic compounds possessed the potent anti-oxidant and anti-cancer activities to a greater or lesser extent [20], moreover, structure–activity relationships for their radical scavenging, anti-oxidant, anti-herbivore, and anti-herpetic activities also had been discussed [21–23]. Here, they were found having a healthy value against cancer or diabetic complications.

Based on our results, the anti-diabetes complications and anti-cancer activity of different parts of *C. mollissina* could be attributed to its containing amount of flavonoids and polyphenolic derivatives especially with more function groups in the structures. It is no doubt that as a food product the value for Ban li is not only for its nutrients but also for its functional values, including the value of its shells and involucres, which would be potential sources of phenolic compounds for using either as food additives or chemical medicines. We believe that systematic and thorough investigations on functions of Chinese chestnut are very necessary in future studies.

## Conclusions

This paper aims to detail some standard procedures to provide better scope for performing the anti-diabetes complications and anti-cancer properties of *C. mollissina*. The plant is thus a promising source of anti-diabetes complications and anti-cancer drug besides indication that flavonoids and polyphenolic acids are the compounds responsible for these effects. Such findings are of extreme importance in the strive for future development of potent, safer and effective anti-diabetes complications and anti-cancer agent.

## Author information

LZ (Lin Zhang) and LZ (Libin Zhan) are associate professor and professor at Dalian medical University, respectively, Academy of Integrative Medicine and the Second Affiliated Hospital. HG and LW are associate professor and professor at Shenyang pharmaceutical University, respectively, Key Laboratory of Structure-Based Drug Design & Discovery of Ministry of Education. MB, YO and TO are associate professor, professor and professor at Meiji Pharmaceutical University, respectively, Department of Natural Medicine and Phytochemistry.

## Electronic supplementary material

Additional file 1: **The 1H-NMR spectrum of compound 6.** (JPEG 41 KB)

Additional file 2: **The 13C-NMR spectrum of compound 6.** (JPEG 47 KB)

Additional file 3: **The 13C-NMR DEPT spectrum of compound 6.** (JPEG 1 MB)

Additional file 4: **The HMBC spectrum of compound 6.** (JPEG 832 KB)

Additional file 5: **The HMQC spectrum of compound 6.** (JPEG 778 KB)

Additional file 6: **The 1H–1H NOESY spectrum of compound 6.** (JPEG 1 MB)

Additional file 7: **The Mass spectrum of compound 6.** (JPEG 72 KB)

## Abbreviations

CK: Alcohol extracts of kernels
CS: Alcohol extracts of shells
CI: Alcohol extracts of involucres
CK-A: EtOAC soluble fractions of CK
CS-A: EtOAC soluble fractions of CS
CI-A: EtOAC soluble fractions of CI
CI-B: The n-BuOH soluble fraction of CI
CK-B: The n-BuOH soluble fractions of CK
CS-B: The n-BuOH soluble fractions of CS
CK-W: The aqueous parts of CK
CS-W: The aqueous parts of CS
CI-W: The aqueous parts of CI
HR-AR: Human recombinant aldose reductase
AGEs: Advanced glycation end products
FBS: Fetal bovine serum
SMPC: Gly steroyl myristoyl phosphatidylcholine glycine
MTT: 3-(4, 5-dimethylthazol-2-yl)-2, 5-diphenyl tetrazolium bromidetetrazolium salt.
