# Supplementary figures and images for: Extracts and compounds with anti-diabetic complications and anti-cancer activity from Castanea mollissina Blume (Chinese chestnut)
Source: BMC Complement Altern Med. 2014 Oct 28;14:422. doi: 10.1186/1472-6882-14-422 (PMC4226895; doi:10.1186/1472-6882-14-422)

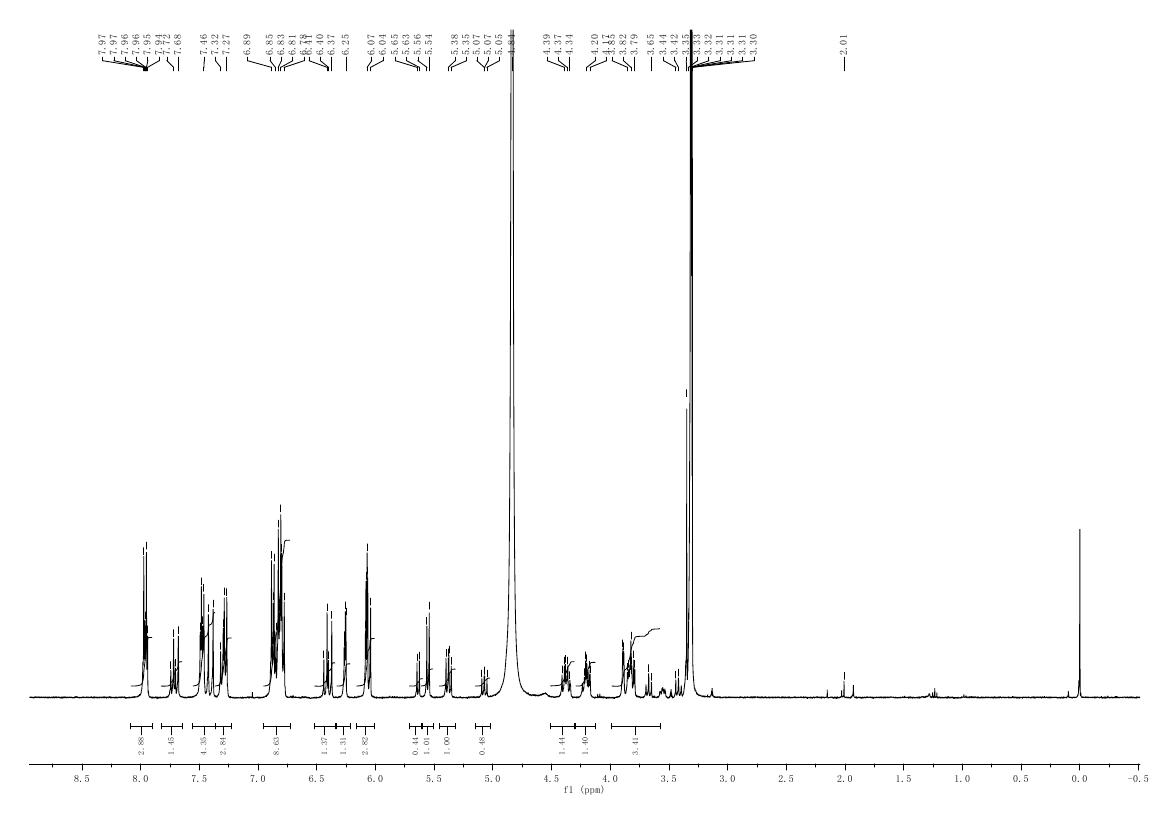

Supplement: Supplementary file 1 — Additional file 1: The 1H-NMR spectrum of compound 6. (JPEG 41 KB) [file 12906_2014_1994_MOESM1_ESM.jpeg]

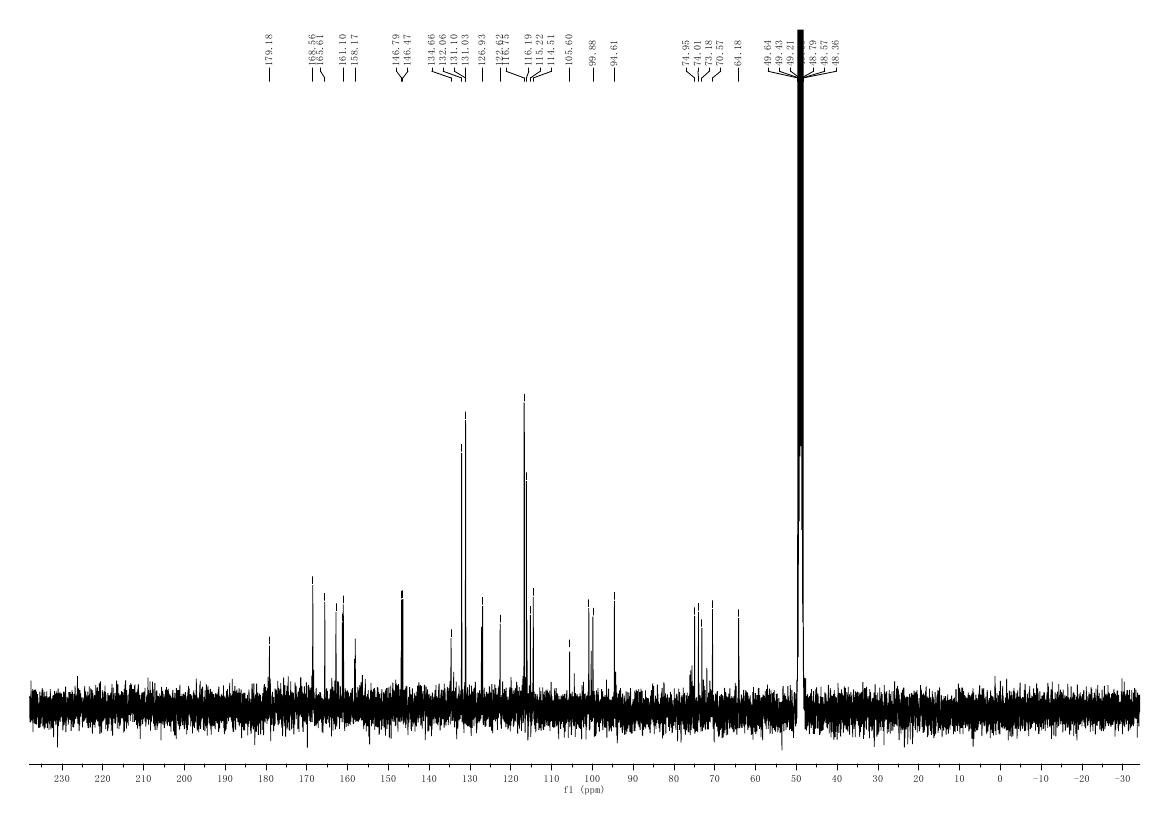

Supplement: Supplementary file 2 — Additional file 2: The 13C-NMR spectrum of compound 6. (JPEG 47 KB) [file 12906_2014_1994_MOESM2_ESM.jpeg]

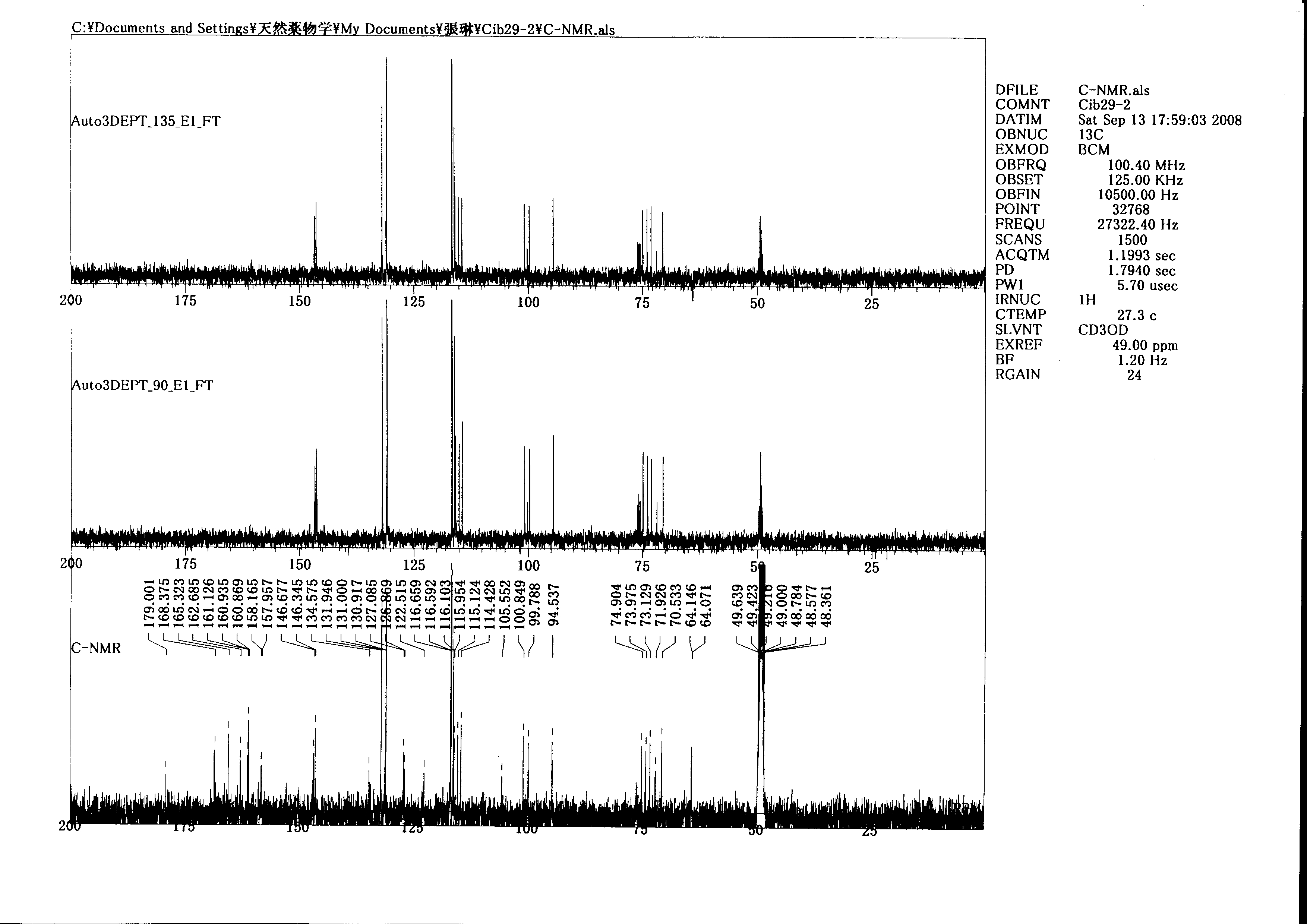

Supplement: Supplementary file 3 — Additional file 3: The 13C-NMR DEPT spectrum of compound 6. (JPEG 1 MB) [file 12906_2014_1994_MOESM3_ESM.jpeg]

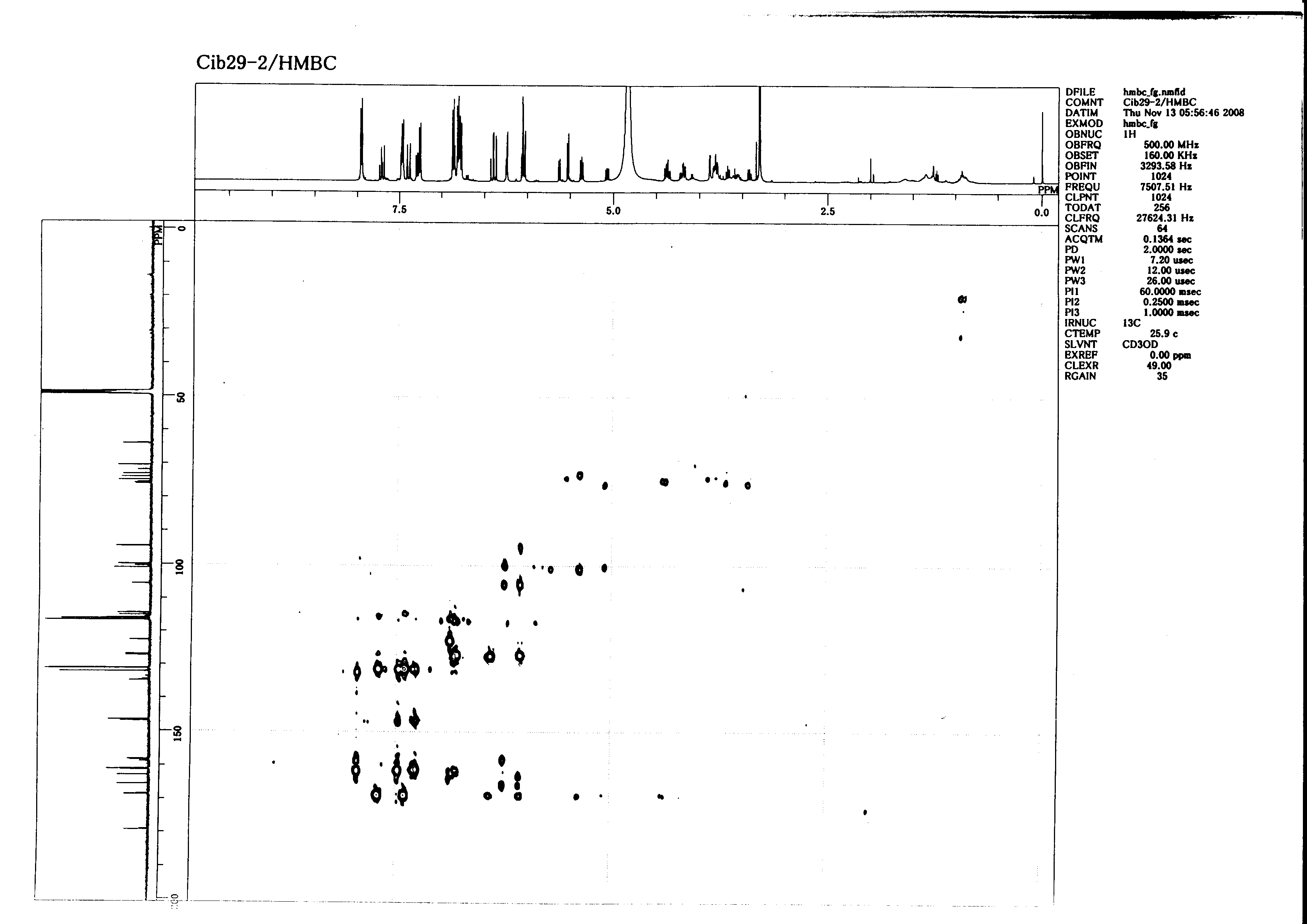

Supplement: Supplementary file 4 — Additional file 4: The HMBC spectrum of compound 6. (JPEG 832 KB) [file 12906_2014_1994_MOESM4_ESM.jpeg]

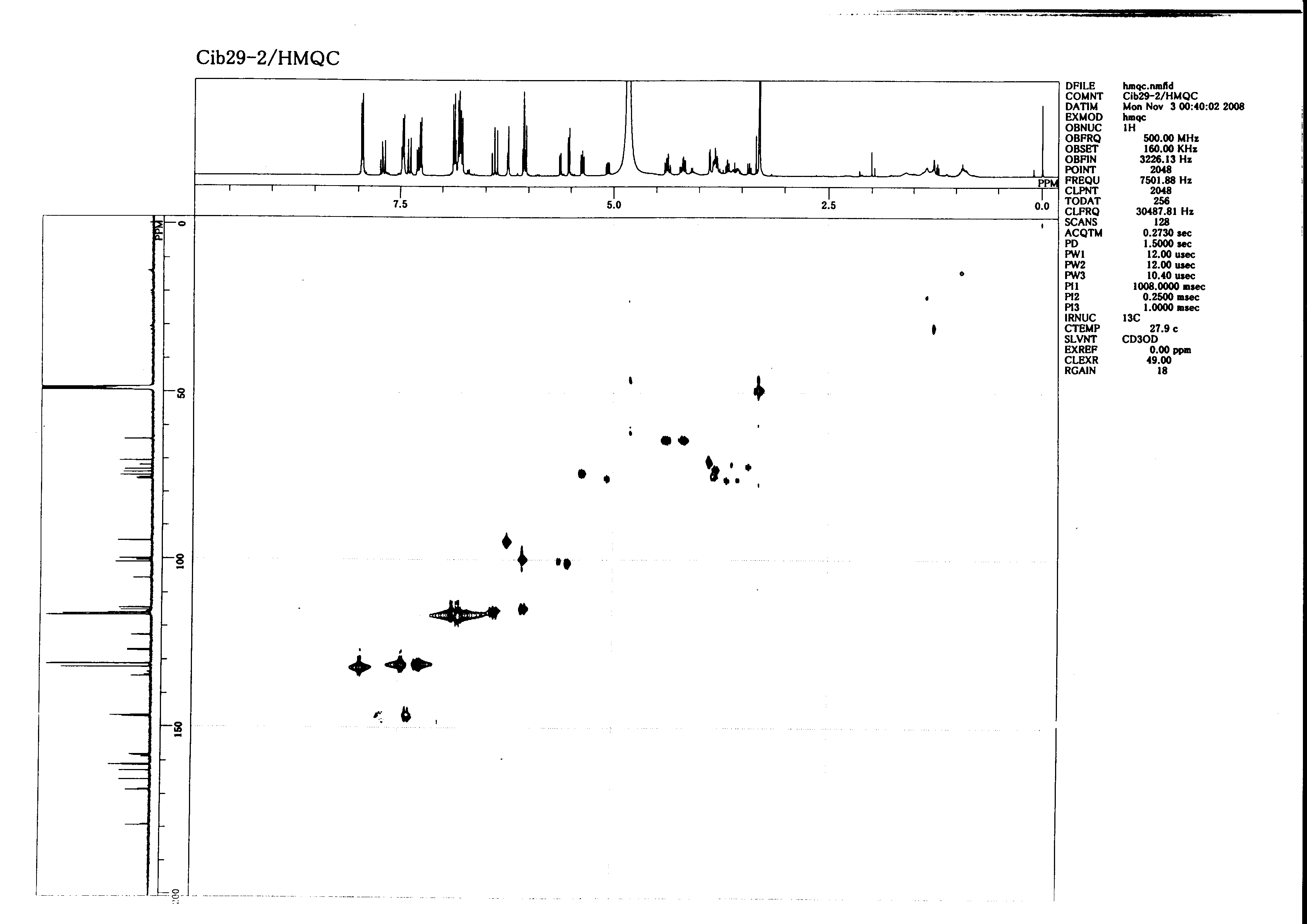

Supplement: Supplementary file 5 — Additional file 5: The HMQC spectrum of compound 6. (JPEG 778 KB) [file 12906_2014_1994_MOESM5_ESM.jpeg]

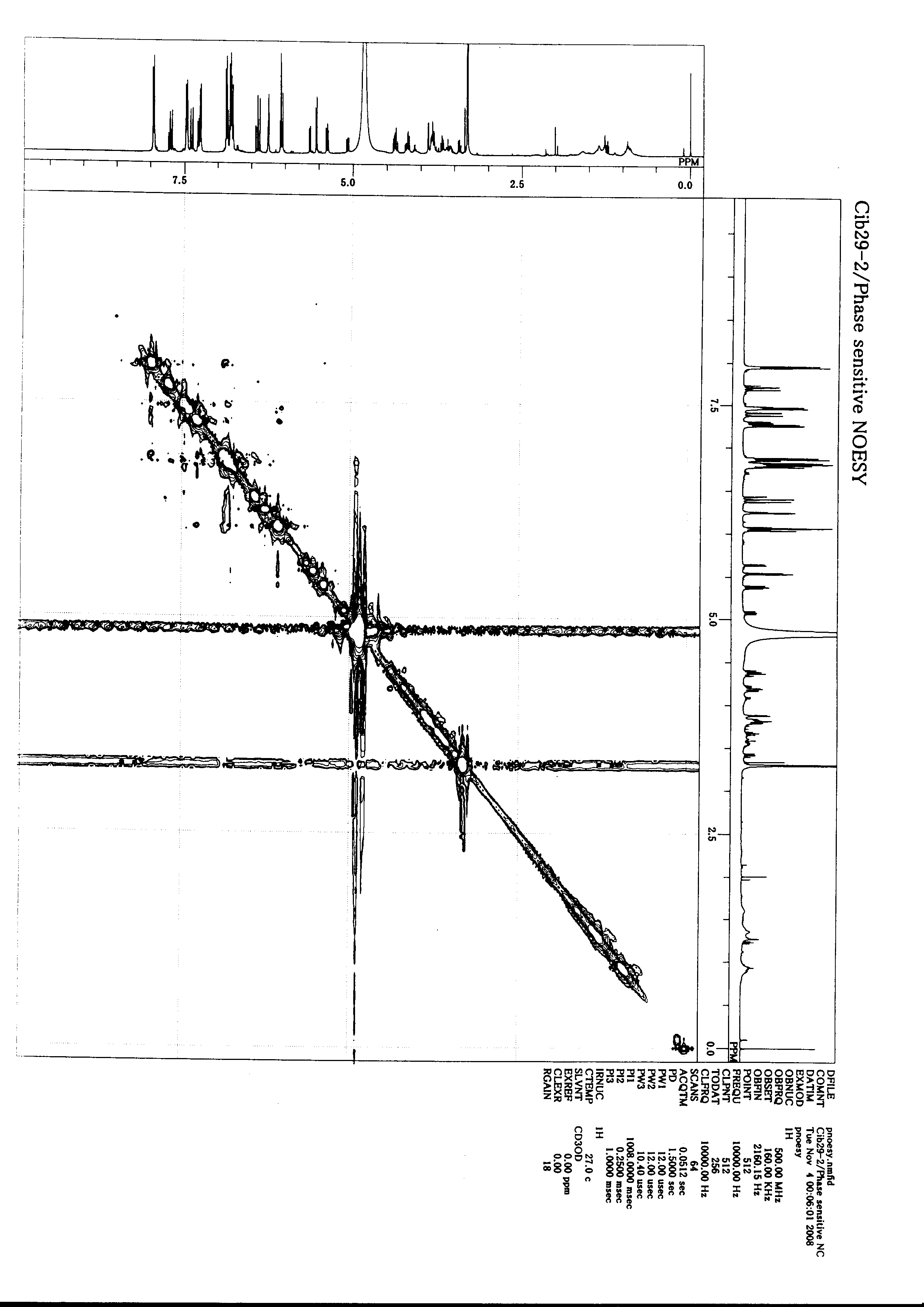

Supplement: Supplementary file 6 — Additional file 6: The 1H–1H NOESY spectrum of compound 6. (JPEG 1 MB) [file 12906_2014_1994_MOESM6_ESM.jpeg]

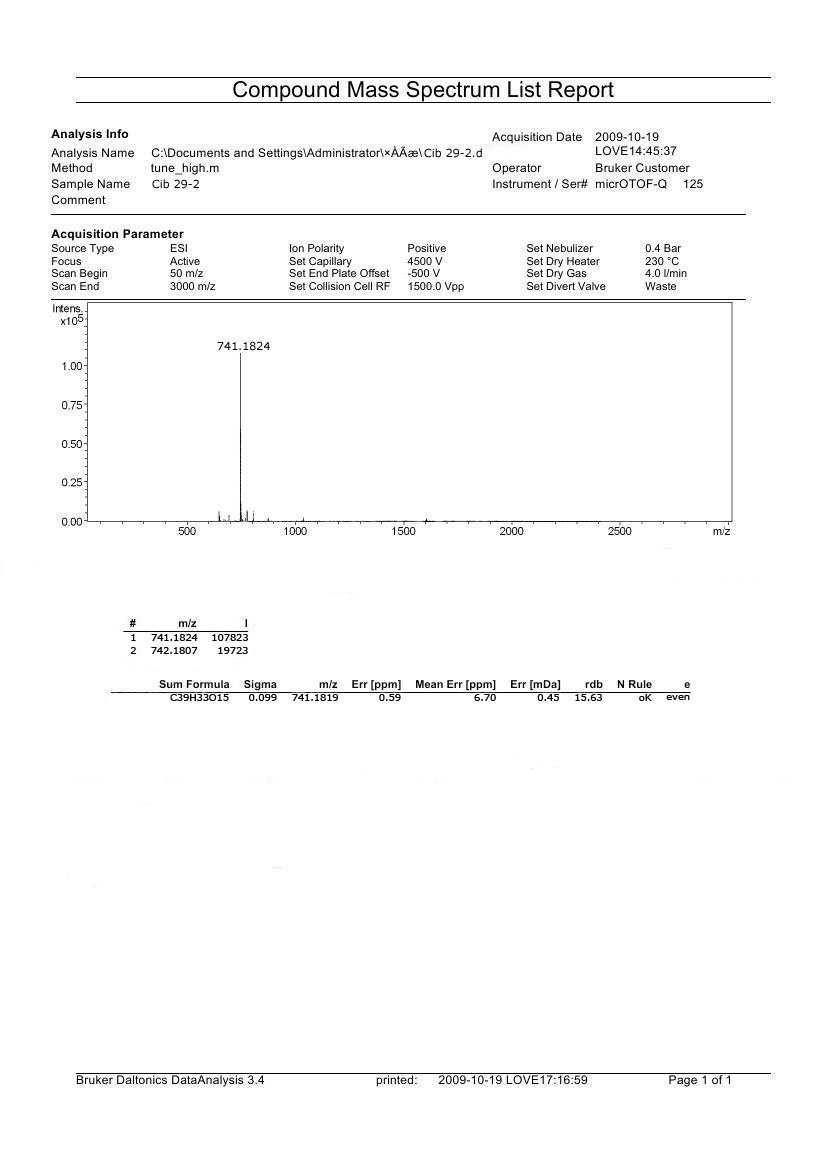

Supplement: Supplementary file 7 — Additional file 7: The Mass spectrum of compound 6. (JPEG 72 KB) [file 12906_2014_1994_MOESM7_ESM.jpeg]
